# Supplementary material for: Integrated into Environmental Biofilm Chromobacterium vaccinii Survives Winter with Support of Bacterial Community
Source: Microorganisms. 2020 Oct 30;8(11):1696. doi: 10.3390/microorganisms8111696 (PMC7716238; doi:10.3390/microorganisms8111696)
Supplement: Supplementary file 1 [file microorganisms-08-01696-s001.zip › Supplementary materials.pdf]

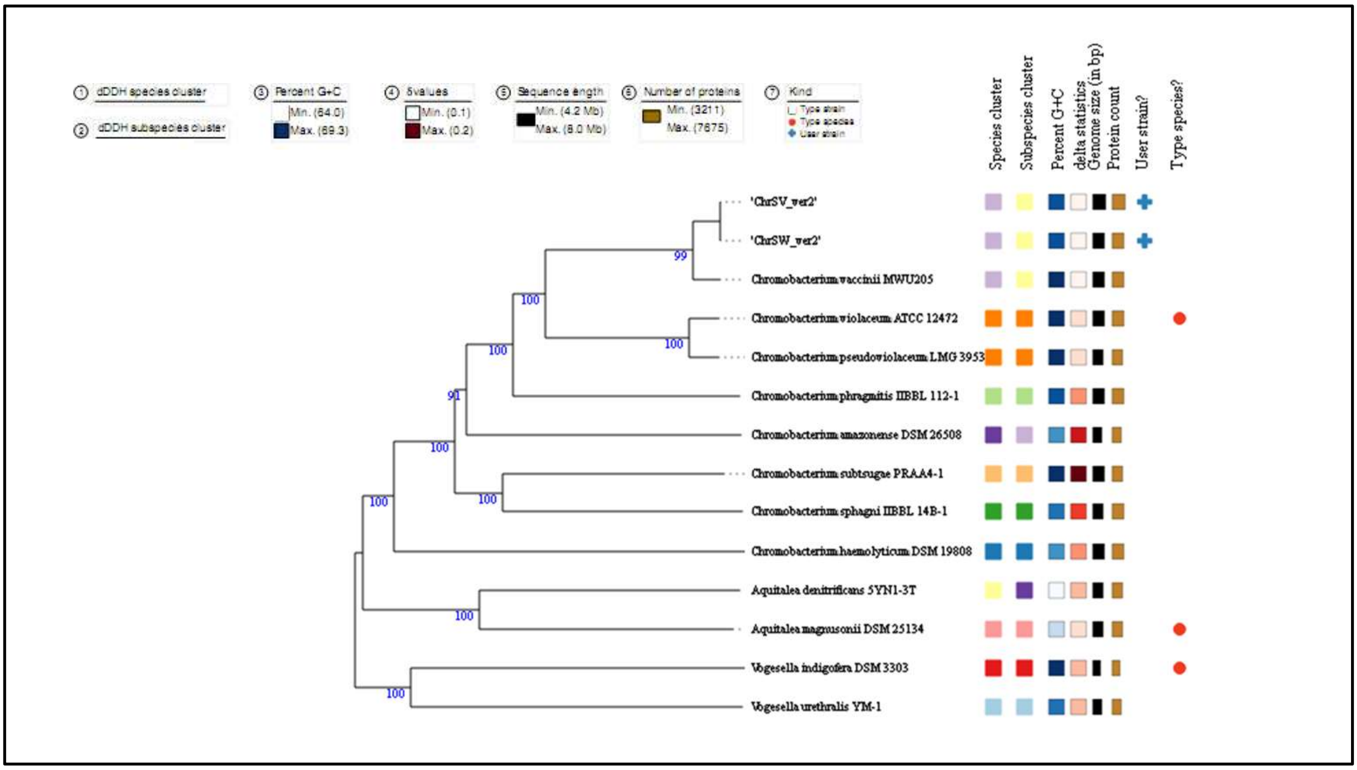

Figure S1. Tree inferred with FastME 2.1.6.1 [Lefort, 2015] from GBDP (Genome BLAST Distance Phylogeny) distances calculated from genome sequences with help of the
Type (Strain) Genome Server [Meier-Kolthoff, 2019].

Lefort V, Desper R, Gascuel O. FastME 2.0: A comprehensive, accurate, and fast distance-based phylogeny inference program. Mol Biol Evol. 2015;32: 2798–2800. DOI: 10.1093/molbev/msv150

Meier-Kolthoff J.P., Göker M. TYGS is an automated high-throughput platform for state-of-the-art genome-based taxonomy. Nat Commun. 2019;10(1):2182. doi: 10.1038/s41467-019-10210-3.

A

Fatty acid	% from total
3OH 10:0	0.30
12:0	12.11
13:0	0.38
2OH 12:0	1.53
3OH 12:0	0.15
14:0	0.16
16:1	0.72
16:0	15.93
17:0	0.10
18:1d9	0.44
18:1d11	0.29
18:0	67.43
20:0	0.42

B

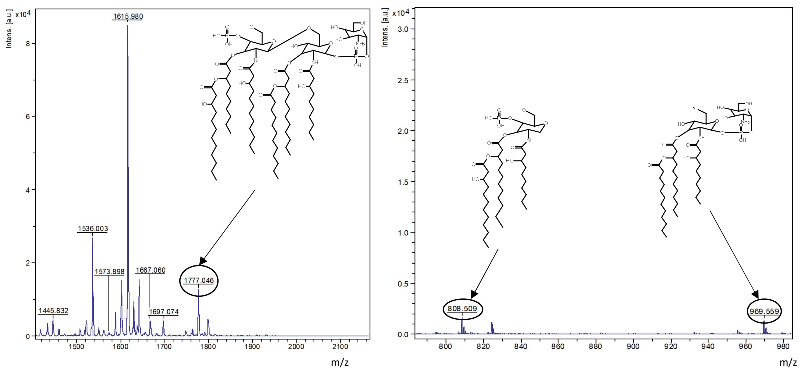

Figure S2. Lipid A structure analysis.

A. Analysis of the fatty acid composition using the methods of gas-liquid chromatography of the extract of lipopolysaccharides makes it possible to determine which fatty acids are acyl groups in the composition of lipid A. However, the extract also contains free fatty acids. **B.** Analysis of the spectra obtained using MALDI mass spectrometry methods allowed to make an assumption about the structure of the lipid A. The peak with an m / z value of 1777.046 (indicated with arrow) corresponds to the deprotonated structure of the putative structure of lipid A. The peaks with m / z values of 808.509 and 969.559 (indicated with arrows) correspond to parts of the proposed structure of lipid A. Also differences in masses corresponds to 3-hydroxydecanoic, 2- or 3-hydroxydodecanoic acid, phospholation and hexoseaminophosphate, that confirmed proposed structure of lipid A.

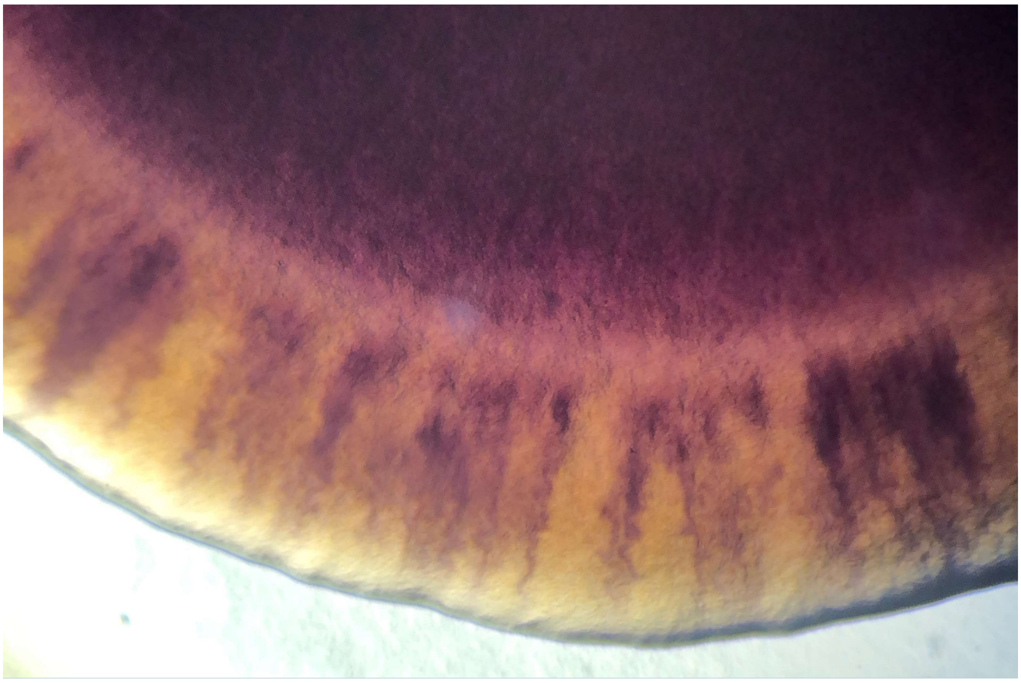

Figure S3. Channel-like structures at the edge of the mixed colony biofilm.
Zip-like structures at the edge of mixed colony biofilm formed by pigmented and unpigmented strains of *C. vaccinii* are similar to the previously described transport intra-colony channels in *E. coli.*

Table S1. Fatty acid composition of several *Chromobacterium vaccinii* isolates.

10:0 3OH	0,20	0,19	0,32	0,33	0,25	0,24	0,21	0,28	0,17	0,13	0,28	0,32
c12	1,30	1,37	1,71	1,71	1,58	1,60	1,32	1,35	1,12	1,01	1,43	1,48
12:0 2OH	0,58	0,55	0,69	0,72	0,56	0,54	0,65	0,73	0,51	0,36	0,69	0,74
12:0 3OH	0,01	0,01	0,04	0,05	0,03	0,03	0,03	0,04	0,01	0,00	0,03	0,03
14:1 w7c	0,18	0,19	0,30	0,30	0,25	0,23	0,22	0,25	0,20	0,15	0,27	0,28
14:1 w5c	0,02	0,03	0,09	0,10	0,02	0,02	0,04	0,06	0,03	0,02	0,08	0,08
c14	3,09	3,11	3,00	3,05	3,09	3,06	2,89	2,96	2,59	2,51	3,15	3,16
15:1 w8c	0,02	0,03	0,11	0,10	0,22	0,20	0,04	0,06	0,06	0,02	0,05	0,05
15:1 w6c	0,17	0,19	0,35	0,36	0,39	0,35	0,29	0,32	0,26	0,21	0,28	0,28
c15	2,29	2,28	2,94	2,98	3,95	3,93	2,45	2,50	2,88	2,79	2,29	2,29
16:1 w5c	44,30	44,17	44,46	44,39	47,52	47,90	43,11	42,99	45,12	45,97	44,51	44,39
c16	33,23	33,19	31,71	31,64	29,37	29,72	33,56	33,07	32,44	33,04	33,95	33,86
17:0 CYCLO	2,37	2,39	1,37	1,35	1,78	1,65	2,94	2,98	1,63	1,47	1,69	1,69
c17	0,18	0,18	0,31	0,31	0,25	0,22	0,27	0,28	0,27	0,25	0,25	0,24
18:1 w7c /12t/9t	10,38	10,45	11,07	11,07	9,09	8,76	10,26	10,23	10,96	10,59	9,36	9,33
c18	0,21	0,22	0,23	0,24	0,13	0,14	0,25	0,27	0,27	0,26	0,24	0,23
